# The protective role of caffeic acid on bovine mammary epithelial cells and the inhibition of growth and biofilm formation of Gram-negative bacteria isolated from clinical mastitis milk

**DOI:** 10.3389/fimmu.2022.1005430

**Published:** 2022-10-20

**Authors:** Tianle Xu, Hao Zhu, Run Liu, Xinyue Wu, Guangjun Chang, Yi Yang, Zhangping Yang

**Affiliations:** ^1^ Joint International Research Laboratory of Agriculture and Agri-Product Safety, Ministry of Education of China, Yangzhou University, Yangzhou, China; ^2^ College of Animal Science and Technology, Yangzhou University, Yangzhou, China; ^3^ College of Veterinary Medicine, Nanjing Agricultural University, Nanjing, China; ^4^ College of Veterinary Medicine, Yangzhou University, Yangzhou, China

**Keywords:** bovine mastitis, gram-negative bacteria, antimicrobial, caffeic acid, anti-inflammatory activity

## Abstract

As a first-line barrier against bacterial infection of mammary tissues, bovine mammary epithelial cells (bMECs) are generally believed to be involved in the immune response due to exogenous stress. Due to the escalating crisis of antibiotic resistance, there is an urgent need for new strategies to combat pathogenic bacteria-infected bovine mastitis. In this study, isolated bMECs and Institute of Cancer Research (ICR) mice were used for *Escherichia coli* infection and caffeic acid (CA) pretreatment experiments *in vitro* and *in vivo*. The inhibitory effect of CA on bacterial growth and biofilm formation was also demonstrated with bacteria strains isolated from mastitis-infected milk. It was demonstrated that CA supplementation prohibits the growth of the predominant strains of bacteria isolated from clinical bovine mastitis milk samples. CA was found to disrupt the biofilm formation of *E. coli* B1 in a sub-minimum inhibitory concentration (sub-MIC) and inhibited the adherence property of *E. coli* on bMECs by decreasing the staining of bacteria on cell surfaces *in vitro*. In addition, CA was found to attenuate proinflammatory and oxidative responses in cells infected with *E. coli*. The pretreatment of bMECs with CA also restored altered lipid homeostasis caused by *E. coli* stimulation. The protective role of CA was further confirmed *via* the administration of CA in mice followed by representative Gram-negative bacterial infection. Collectively, these findings highlight the potential of CA to mediate Gram-negative infections and indicate that it has the potential to be developed as a novel antibacterial drug.

## Introduction

Bovine mastitis is characterized by the invasion and colonization of bacteria in mammary tissue, threatening dairy production and milk quality (1–3). Clinical mastitis infection by *Escherichia coli* or *Klebsiella pneumoniae* is environmentally linked, as compared to contagious pathogens such as *Staphylococcus aureus* and *Streptococcus agalactiae* (4, 5). Excessive inflammation induced by toxins secreted from these pathogens leads to pathological courses such as lesions on the mammary tissue, which impair the cow’s performance as a milk producer (6). It has been reported that endotoxins including lipopolysaccharide (LPS), the component that envelops the wall of Gram-negative bacteria, can induce a storm of inflammatory responses in bovine mammary epithelial cells (bMECs) (7, 8). This kind of induction of inflammation appears to elicit several sets of innate immune response, oxidative stress, and metabolic reprogramming-related genes (9, 10). Epidemiological investigations reveal that Gram-negative bacteria, such as *E. coli* and *K. pneumoniae*, in bovine mastitis are becoming more common on dairy farms worldwide and that pathogens are becoming multidrug-resistant (MDR) (11, 12).

Along with therapeutic strategies, such as antibiotic therapy, epithelial cells act as a first barrier against invading bacteria and as a bridge for the host’s defense response (13, 14). bMECs are involved not only in the production of milk but also in immune regulation stimulated by exogenous pathogens (15–17). During *E. coli* mastitis, often-generalized inflammatory responses are regulated by the number of immune molecules, such as toll-like receptor 4 and the NF-κB complex transcription factor (18, 19). These, in turn, act as a master switch to activate a wealth of proinflammatory genes (20). Moreover, the defensive process of bMECs has also been suggested to elicit oxidative damage and metabolic changes in response to *E. coli* infections (21). Eradicating bacteria at an earlier time is of great importance to control the inflammation of epithelial cells and restore tissue homeostasis (22). Epithelial cells usually regulate multiple defensive mechanisms against bacterial invasion *via* alteration of cell integrity, apoptosis, and autophagy (22, 23). For MDR bacteria, a prolonged attachment to cells provides opportunities for the invasion to remain viable and remain in the cytosol of epithelial cells (24). With respect to the persistent drug resistance of pathogens related to bovine mastitis, antibiotics are still effective in clearing bacteria, but overuse or misuse leads to the problems such as MDR, antibiotic tolerance, and lack of food safety (25–27). Therefore, it is urgent to develop alternative strategies to antibiotics in the treatment of MDR bacteria-infected bovine mastitis.

Caffeic acid (3,4-dihydroxycinnamic acid; CA) is a phenolic compound that is present in several plants (28). It has been employed as a strategy for combating pathogenic bacteria or chronic infection due to its antimicrobial and anti-inflammatory activities, as well as properties related to boosting immunity (29). To uncover the protective role of CA in bMECs, a study revealed that CA suppresses LPS-induced inflammatory responses *via* the suppression of NF-κB/MAPK cascades (30). However, being predominantly biologically active, caffeic acid might also participate in antioxidation and metabolic homeostasis in bMECs, as well as interactions between pathogens and host-cell immune responses, but these qualities remain largely unknown. The present study investigated the role of CA in alleviating *E. coli*-induced detrimental effects in bMECs with regard to the inhibition of inflammation responses, oxidative stress, and alteration of fatty acid metabolism. Moreover, reductions in Gram-negative bacteria growth and the internalization properties of bMECs were also characterized, as well as the amelioration of damage in mouse mammary glands. The results may provide insights into the exploitation of strategies against Gram-negative pathogen-induced bovine mastitis, as well as the treatment for homologous infectious diseases.

## Materials and methods

### Ethics

The experimental protocols were verified by the Animal Experiment Committee of Yangzhou University according to the Regulations for the Administration of Affairs Concerning Experimental Animals in China. The experiments were strictly implemented in accordance with the approved guidelines and regulations (YZU-2022002-096).

### Cell culture conditions

Primary bMEC cultures were performed as described previously (21, 31). In brief, three Holstein cows in mid-lactation (30.26 ± 3.1 kg/day of milk production, 2.7 parity, and 175 ± 6 days in milk) with absence of clinical incidence of diseases were employed for isolation of cells. The bMECs were purified by differential centrifugation, and the digested solution containing fibroblasts was discarded after trypsin digestion for 3 min, and then the culture medium was added to continue the cell culture. Cell passage from the fourth to sixth generation was used in the current study; 2 × 10^5^ of cells were seeded in six-well plates with medium containing Roswell Park Memorial Institute (RPMI) with 10% heat-inactivated fetal bovine serum (Gibco, San Diego, CA, USA) and antibiotics (penicillin 100 IU/ml; streptomycin 100 μg/ml) (Thermo Fisher Scientific, Pleasanton, CA, USA). The incubation of cells was maintained at 37°C with a 5% CO_2_ condition.

### Experimental design

#### Cell experiment

The *E. coli*-induced inflammatory model was established and described previously (32). The optimized CA doses in the current study were selected according to a previous study, with few modifications (30). Bacteria used in the infection experiment were quantified by surviving bacteria counting method (colony-forming unit (CFU) counting). No toxicity of CA treatment to cells was found at concentrations from 10 to 500 μg/ml *via* the cell viability test. Cells stimulated with *E. coli* (1 × 10^7^ CFU/ml) for 6 h were selected as the positive control (*E. coli*); cells pretreated with CA at concentrations of either 125 or 250 μg/ml for 12 h, followed by the *E. coli* (1 × 10^7^ CFU/ml) challenge for 6 h (C125+E; C250+E). Cells treated with phosphate-buffered saline (PBS) for 18 h were selected as negative control (NC).

#### Bacteria and reagents

All strains were isolated from the milk of dairy cattle with clinical signs of mastitis, which were validated by the California Mastitis Test (CMT) according to the recommendation of the U.S. National Mastitis Council (33, 34). The isolation and identification of all strains used in the current study are described in the **Supplementary Material**. The pathogenicities of isolated *E. coli*, *S. aureus*, *Pseudomonas aeruginosa*, and *K. pneumoniae* strains in mice have been determined, and results are indicative of the serious infection with histological changes in tissues like the liver and spleen. Unless otherwise noted, bacteria were grown in Mueller–Hinton broth (MHB; HB6231, Qingdao Hope Bio-technology) or on MH agar (MHA, HB6232) plates at 37°C. Caffeic acid with a purity of more than 98% was purchased from Sigma-Aldrich (Sigma, St. Louis, MO, USA; C0625).

#### Animal experiment

Six to 8-week-old male Institute of Cancer Research (ICR) mice and 1-week lactating female ICR mice were obtained from the Center of Comparative Medicine at Yangzhou University. One week prior, infection was set for adaption of mice to the standard environment conditions (23°C ± 2°C; 55% ± 10% of humidity). The experimental design was described as follows: male ICR mice (70 in total) were separated into seven groups, with each group comprising 10 mice:

1. Control group: mice normally treated were taken as control.
2. Bacteria challenge group: mice received an intraperitoneal injection with *E. coli* YZ6 or *K. pneumoniae* SH1 (1 × 10^8^ CFU/ml) on day 11 for 6 h.
3. CA + bacteria group: mice were orally administrated with CA at a dose of 25 or 50 mg/kg/day for 10 days, following *E. coli* or *K. pneumoniae* challenge at day 11 for 6 h.

Lactating ICR female mice (40 in total) were separated into four groups with each group comprising 10 mice:

1. Control group: mice normally treated were taken as control.
2. *E. coli* challenge group: mice received intramammary injection with *E. coli* B1 (1 × 10^6^ CFU/ml) on day 11 for 6 h.
3. CA + *E. coli* group: mice were orally administrated with CA at a dose of 25 or 50 mg/kg/day for 10 days, followed by *E. coli* intramammary injection on day 11 for 6 h.

Animals were placed under anesthesia at 6 h after pathogenic injection and sacrificed by cervical dislocation. The doses of CA at 25 or 50 mg/kg were used in this study based on the acute toxicity test with pathological observation of animals treated with CA at a range of 10 to 100 mg/kg. Anticoagulation tubes (EDTA) were used for collecting the peripheral blood from the removed eyeballs. Mammary tissues were excised and rinsed in cold PBS, and all the samples were stored at −80°C immediately after collection.

### Lethality assessment

The mortality rates were determined for a duration of 24 h in groups as selected in the present study. The time points for monitoring the death of mice were 8 h, 12 h, 18 h, and 24 h post-injection of bacteria.

### Histological determination

Pieces of mammary tissues sampled from all experimental groups were fixed in buffered formalin phosphate (10%). Then, fixed samples were paraffin-embedded, sectioned, and stained with hematoxylin and eosin, and a phase-contrast microscope was used for imaging (Nikon, Tokyo, Japan).

### Peripheral malondialdehyde content

The concentration of malondialdehyde (MDA) in peripheral plasma from all groups was determined by ELISA commercial kit and performed in accordance with the manufacturer’s instruction (Jiancheng Bioengineering Institute, Nanjing, China).

### Minimum inhibitory concentration and minimum bactericidal concentration determination

The determination of minimum inhibitory concentration (MIC) for CA on each bacterial strain was performed using the standard microdilution approach recommended by the Clinical and Laboratory Standards Institute (CLSI) (2017). Briefly, a twofold dilution of CA with an equal volume of bacterial cultures containing 1.5 × 10^6^ CFU/ml in a 96-well microliter plate is available. The MIC values were recognized as the lowest concentrations of the CA with invisible bacteria growth. For minimum bactericidal concentration (MBC) of CA, bacteria cultures containing CA equal to or higher than MIC were streaked on blood agar plates and incubated at 37°C for 24 h. The concentration of CA at which no visible colony was observed on the plate after 24-h incubation was defined as MBC.

### Bacteria growth curve determination

The overnight bacterial culture at 37°C from a single colony was prepared and diluted at 1:10,000 into an MHB medium. CA at concentration of 0, 0.5625, 1.25, 2.5, and 5 μg/ml was added to the cultures. The optical density at OD_600nm_ was measured by a spectrophotometer (Tecan, Spark, Switzerland) at 0, 4, 6, 8, 12, and 24 h of incubation. Experiments were performed in triplicate.

### Biofilm morphology assay

CA was diluted to 0.5× MIC, 1× MIC, 2× MIC, and 4× MIC in MHB medium. The overnight culture was added to a six-well plate placed with crawling slides for 24-h incubation at 37°C without shaking. For live or dead cell determination in matured biofilm, the 24-h incubated biofilm was added with or without CA for an additional 24 h. The slides were washed with PBS to remove planktonic cells for staining with SYTO9 (Green) and PI (Red) pre-mixture using LIVE/DEAD BacLight Bacterial Viability Kit (L7007, Thermo Fisher Scientific, Waltham, MA, USA). The images were captured using DMi8 Microsystems BmbH (Leica, Wetzlar, Germany). For the biofilm formation inhibition experiment, 40 μl of overnight culture was seeded to a six-well plate containing 1,960 μl of diluted CA. Cells were stained with 1.5% Cresyl Violet (CV) and visualized using DMi8 Microsystems BmbH (Leica, Wetzlar, Germany).

### Scanning of bacteria adhesion

For scanning the level of *E. coli* adhesion, cell sample nuclei were counterstained with Hoechst 33342 (Beyotime, Beijing, China). The bacterial was pHrodo green probes labeled prior to the infection of bMECs (Thermo Scientific, MA); 1 × 10^6^ CFU bacteria and increasing doses of CA at 75, 125, and 250 μg/ml were cocultured with bMECs at 37°C for 3 h. The images were captured using DMi8 Microsystems BmbH (Leica, Wetzlar, Germany).

### Counting for bacteria adhesion to bovine mammary epithelial cell

Cells were seeded on the 12-well plates until the growth of 90% prior to the bacteria challenge 1 × 10^6^ CFU bacteria and increasing doses of CA at 75, 125, and 250 μg/ml were cocultured with bMECs at 37°C for 3 h. The cells were washed three times with PBS to remove the planktonic cells and re-suspended with trypsin. Cocultured suspension was spread on an MH agar plate with 10 times’ dilution. The final CFUs were determined by counting the colonies after incubation at 37°C for 18 h.

### 5-Ethynyl-2′-deoxyuridine determination

The BeyoClick 5-ethynyl-2′-deoxyuridine (EdU) cell proliferation kit with Alexa Fluor 555 (Beyotime, Shanghai, China) was applied for the determination of cell proliferation under different treatments and performed as described previously (35). EdU solution with a concentration of 10 μM was used to incubate cells for 2 h; 4% paraformaldehyde was used for fixation, and 0.3% Triton X-100 was used to improve the permeabilization of the cells. The nuclei of cells were stained using Hoechst 33342 for 10-min incubation at room temperature (RT) free of light. The proliferation rate was assessed by imaging with a fluorescence microscope DMi8 Microsystems GmbH (Leica, Wetzlar, Germany).

### Cell migration assay

The scratch-wound assay was used for evaluating the migration of bMEC. Confluent cells grown in a six-well plate were linearly scratched using a micropipette tip as selected treatments. The interval gap of the cells was monitored at 0, 12, and 24 h after scratching. A phase-contrast microscope was used for capturing images (Nikon, Tokyo, Japan). ImageJ software was used for evaluating the wound closure rate (LOCI). Experiments were performed in triplicate.

### ELISA for cytokine analysis

For the experiment *in vitro*, the level of TNF-α and IL-6 in the cultured medium was measured by ELISA, which was conducted using the DuoSet ELISA bovine IL-6 (R&D Systems, Minneapolis, MN, USA) and DuoSet ELISA bovine TNF-α (R&D Systems, Minneapolis, MN, USA) kits in accordance with the manufacturer’s instructions.

For the experiment *in vivo*, contents of TNF-α, IL-1β, and IL-6 in murine mammary gland were quantified by using a commercial Quantikine ELISA kit for mice (MLB00C, MTA00B, and M6000B, respectively, R&D Systems, Minneapolis, MN, USA) according to the manufacturer’s instructions. The evaluation reports for the sensitivity and specificity of these kits could be found at https://www.rndsystems.com/.

### Reactive oxygen species, malondialdehyde, glutathione peroxidase, and total antioxidative capacity determination

The level of reactive oxygen species (ROS) production in all groups of cells was determined using an intracellular staining kit that contains labeled 2′,7′-dichlorofluorescein diacetate (DCFH-DA) (Beyotime, Shanghai, China) and was captured by DMi8 Microsystems BmbH (Leica, Wetzlar, Germany). MDA, glutathione peroxidase (GSH-Px), and total antioxidative capacity (T-AOC) were determined at 532, 420, and 520 nm of absorbance, respectively. The results were recorded by using a spectrophotometric diagnostic kit according to the manufacturer’s instructions (Jiancheng Technology, Nanjing, China).

### Oil red O staining

The formation of lipid droplets was stained by oil red O using the commercial kit (C0157S, Beyotime, Beijing, China). Briefly, cells grown in a 12-well plate with crawling slides were treated according to the experiment design. The crawling slides were dried and stained with oil red O dye at RT for 30 min. The cell nucleus was dyed dark blue, and the other section of the cell was dyed light blue using Hematoxylin Staining Solution (C0107B, Beyotime, Beijing, China). A phase-contrast microscope was used for capturing images (Nikon, Tokyo, Japan).

### RNA extraction and qRT-PCR

Total RNA was extracted from cells using an RNA-easy Isolation Reagent (Vazyme, Nanjing, China) according to the manufacturer’s instruction. The obtained RNA quality was assessed using a 2100 bioanalyzer (Agilent Technologies, Santa Clara, CA, USA), and the RNA quality number (RQN) was >7.0 for all samples. cDNA was synthesized by HiScript III RT SuperMix (R323-01, Vazyme, China), and AceQuniversal SYBR Master Mix for qPCR was used in the qRT-PCR experiment (Vazyme, China) on an ABI QuantStudio PCR system (Applied Biosystems, Foster City, CA, USA). Primers involved in the present study were described previously (36, 37). Internal control genes including GAPDH, UXT, and RPS9 were selected for normalization of each target gene’s expression by using geometric mean. The selection of the internal genes was illustrated in bMECs by previous publications (38). The 2^−ΔΔCt^ approach was utilized for relative quantification (39).

### Western blotting

Western blotting was conducted as previously described (10). Briefly, extracted total protein from bMECs using radioimmunoprecipitation assay (RIPA) lysis buffer (Beyotime, Shanghai, China) was quantified with bicinchoninic acid (BCA) measurement (Pierce, Rockford, IL, USA). The protein was adjusted to an equal amount for the separation on 4%–20% sodium dodecyl sulfate (SDS)–polyacrylamide gels. The separated proteins were then transferred onto nitrocellulose-made membranes (Millipore, Billerica, MA, USA) and incubated with target primary antibodies (Cell Signaling Technology (CST), Danvers, MA, USA) overnight at 4°C on the rotator. The primary antibodies for phospho-p65, p65, TLR4, MYD88, Nrf2, SOD1, SOD2, Gpx1, PPARγ, FASN, SCD, CPT1α, and internal reference (GAPDH) were commercially obtained from Cell Signaling Technology (#3033, #8242, #14358, #4283, #12721, #37385, #13141, #3286, #2435, #3180, #2794, #97361, and #5174) and diluted as 1:1,000. Primary antibodies for SREBP1 and PPARα were obtained commercially from Abcam (Cambridge, UK; ab28481, ab126285) and diluted as 1:2,000. The blots were incubated with horseradish peroxidase-coupled (HRP) secondary antibodies with a dilution of 5,000 (#7074, CST) after moderate washing with TBST. The intensity of GAPDH was taken as normalization of each blot for quantification. The blots were analyzed and quantified by using ImageJ software (LOCI).

### Immunofluorescence observation

Cells were seeded onto a crawling slide (φ20mm) installed with a 12-well plate; 4% paraformaldehyde was used to fix cells for 15 min after the selected treatments, and the wells were washed three times with PBS and incubated with Triton-X (0.5%) for 15 min at RT. Slides were then blocked by incubating with bovine serum albumin (BSA) (5%) at 37°C for 1 h. A primary antibody (targeting the p65) was used to incubate cells at 4°C overnight. Fluorescein isothiocyanate (FITC)-labeled secondary antibody was used for staining the cells. After three times washing with PBS, DAPI (1 μg/ml, D8417, Sigma-Aldrich) was used for staining of nuclei for 5 min at RT. DMi8 Microsystems BmbH was used for capturing images (Leica, Wetzlar, Germany).

### Statistical analysis

mRNA and protein expression data were log-2 back-transformed for ease of interpretation and reported in the figures. Data were analyzed using one-way ANOVA with Dunnett’s *post-hoc* test by SAS Statistics (v. 9.2, SAS Institute Inc., Cary, NC, USA). Data were expressed as means ± SEM. *p*-Values <0.05 were considered statistically significant differences. Experiments were performed in triplicate.

## Results

### Bactericidal effect of caffeic acid on Gram-negative bacteria

To evaluate the inhibitory effect of CA on pathogens derived from bovine mastitis, we selected isolates from the clinical and standard strains for MIC and MBC determination, the results of which are listed in **Table 1**. The MIC of CA against *E. coli* strains ranged from 250 to 500 μg/ml, and the MBC was 500 μg/ml. For *K. pneumoniae* strains, the MIC of CA was 500 μg/ml, whereas the MBC was from 500 to 1000 μg/ml. *P. aeruginosa* strains showed less sensitivity to CA, as compared to other Gram-negative bacteria, as a result of MIC being at 1,000 mg/ml and MBC being at 1,000 to 2,000 μg/ml. As bacteria that induce bovine mastitis, *S. aureus* strains presented a similar sensitivity to CA, with MIC and MBC ranging from 250 to 500 μg/ml.

**Table 1:** MIC and MBC of CA for clinical isolated Gram-positive *S. aureus* and gram-negative *Escherichia coli*, *Klebsiella pneumoniae*, and *Pseudomonas aeruginosa*.

Strains	Type	MIC (μg/ml)^a^	MBC (μg/ml)^b^
*E. coli*	ATCC 25922	250	500
*E. coli SH2*	Clinical isolate (A)	250	500
*E. coli YZ6*	Clinical isolate (B1)	250	500
*E. coli SH16*	Clinical isolate (B2)	250	500
*E. coli XY25*	Clinical isolate (D)	500	500
*K. pneumoniae*	ATCC 4352	500	1,000
*K. pneumoniae SH1*	Clinical isolate (ST2661)	250	500
*K. pneumoniae XY2*	Clinical isolate (ST43)	500	500
*K. pneumoniae SH5*	Clinical isolate (ST2410)	500	1,000
*K. pneumoniae YZ6*	Clinical isolate (ST1256)	500	1,000
*P. aeruginosa PAO1*	ATCC 15692	1,000	1,000
*P. aeruginosa HA11*	Clinical isolate (ST277)	1,000	1,000
*P. aeruginosa HA21*	Clinical isolate (ST450)	1,000	2,000
*P. aeruginosa HA8*	Clinical isolate (ST571)	1,000	2,000
*S. aureus*	*ATCC 25923*	250	500
*S. aureus (MRSA)*	*Clinical isolate* (*ST188*)	250	500
*Staphylococcus haemolyticus*	*Clinical isolate* (*ST77*)	500	500
*Staphylococcus chromogenes*	*Clinical isolate* (*ST51*)	500	500

CA, caffeic acid; MRSA, methicillin-resistant S. aureus.

a Minimum inhibitory concentration.

b Minimum bactericidal concentration.

**Figure 1:**
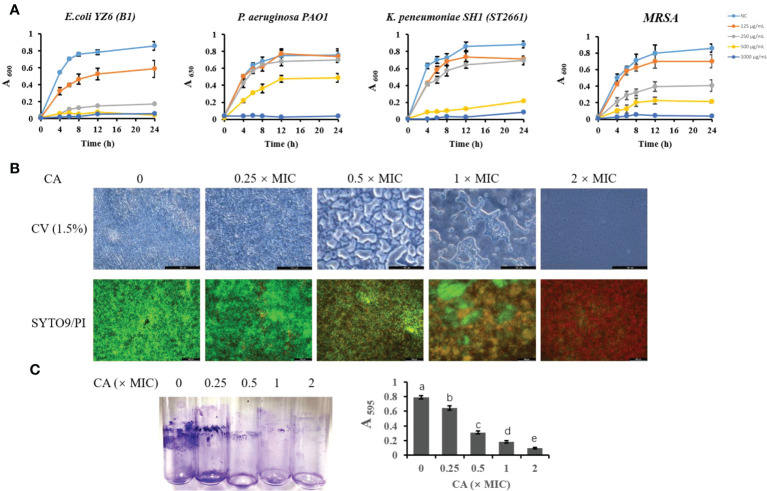
The inhibitory effect of CA on bacterial growth and *Escherichia coli* biofilm formation. **(A)** Effect of CA supplementation on growth of *E. coli* B1, PAO1, *Klebsiella pneumoniae*, and MRSA in LB medium for 24h. **(B)** The effect of CA on *E. coli* B1 biofilm formation. The inhibitory effect of CA on biofilm growth was determined using CV (1.5%) staining, while eradication of matured biofilm by CA was determined using SYTO9 (green) and PI (red) staining. **(C)** Biomass of biofilm formation was determined in tubes and quantified using the absorbance measured at 595 nm. The letters in superscript indicate that the difference between groups was significant (*p* < 0.05). Data are presented as mean ± SEM. The results are representative of three independent experiments. CA, caffeic acid; MRSA, methicillin-resistant *Staphylococcus aureus*; LB, Luria broth; CV, Cresyl Violet; PI, propidium iodide.

To further validate the antibacterial activity of CA, we tested the most sensitive types (lower MIC and MBC) of *E. coli*, *K. pneumoniae*, *P. aeruginosa*, and *S. aureus* strains isolated from milk (**Figure 1A**). The growth curve showed that *E. coli* B1 did not grow at concentrations of CA higher than 250 μg/ml, while 0.5× MIC (125 μg/ml) did not eliminate the activity of *E. coli* B1 as compared to the higher doses. Concentrations of CA less than 500 μg/ml exhibited no effect on bacterial activity in the coculture of *P. aeruginosa* PAO1. Consistently, no values were observed at 1,000 μg/ml of CA, which is consistent with the results of MIC on *P. aeruginosa* PAO1. The concentration of CA at 500 μg/ml significantly blocked the growth of *K. pneumoniae* SH1 (ST2661). Notably, the inhibitory effect of CA on Gram-positive methicillin-resistant *Staphylococcus aureus* (MRSA) appeared to be dose-dependent during a 24-h coculture. In summary, these results demonstrate the potential antibacterial efficacy of CA against pathogenic bacteria, particularly *E. coli* B1.

### Caffeic acid disrupted the biofilm formation of *Escherichia coli* B1

The biofilm formation of bacteria is significantly relevant to increases in drug resistance. CV staining is applied to not only bacteria but also the extracellular matrices of biofilms. In the present study, an increase in CA concentration gradually decreased the biomass of biofilm in the *E. coli* B1 strain (**Figures 1B, C**). Notably, when the concentration of CA was at 2× MIC (500 μg/ml), biofilm formation was completely inhibited. A LIVE/DEAD staining assay showed that an increased CA concentration eradicated mature biofilm as a result of the decreased viability of cells. The concentration of CA at 2× MIC (500 μg/ml) completely eliminated cells in the biofilm matrix, which is consistent with the use of MBC on *E. coli* B1.

### Caffeic acid reduced *Escherichia coli* adhesion of host epithelial cells

**Figure 2:**
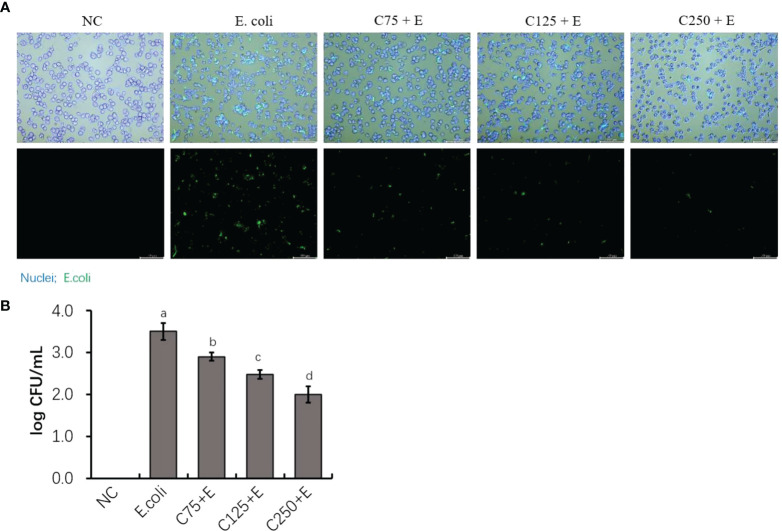
The inhibition of adherence property of *Escherichia coli* B1 in bMECs by CA supplementation. **(A)** Image of bacterial adhesion in bMEC. Cells were infected with bacteria for 3 h, along with supplementation of CA at doses of 75, 125, and 250 μg/ml. Nuclei were counterstained with Hoechst 33342 (blue), while *E. coli* were labeled with pHrodo (green). Scale bar = 100 μm. **(B)** Counting for adhered bacteria in bMEC. Cells were seeded on the 12-well plates, and 1 × 10^6^ colony-forming unit (CFU) bacteria and increasing doses of CA at 75, 125, and 250 μg/ml were cocultured with bMECs at 37°C for 3h. The final CFUs were determined by counting the colonies after incubation at 37°C for 18.h. Data are presented as mean ± SEM. The results are representative of three independent experiments. The letters in superscript indicate that the difference between groups was significant (*p* < 0.05). Data are representative of three independent replicates. bMECs, bovine mammary epithelial cells; CA, caffeic acid.

To determine the adhesion activity of *E. coli* B1 and the inhibitory effect of CA on the adhesion of *E. coli* in bMECs, in the present study, we determined the adhered bacterial counts. As shown in **Figure 2A**, pHrodo green-probed *E. coli* adhered to bMECs with a strong stain, while CA inhibited the adhesion of *E. coli* after the amount added to the cocultured medium was increased. Counting bacteria for adhesion activity consistently showed that an increased amount of CA significantly reduced the adhesion on bMECs with a decreased CFU plated on an MH agar plate (**Figure 2B**).

### Impeded cell proliferation was recovered by supplementation with caffeic acid

**Figure 3:**
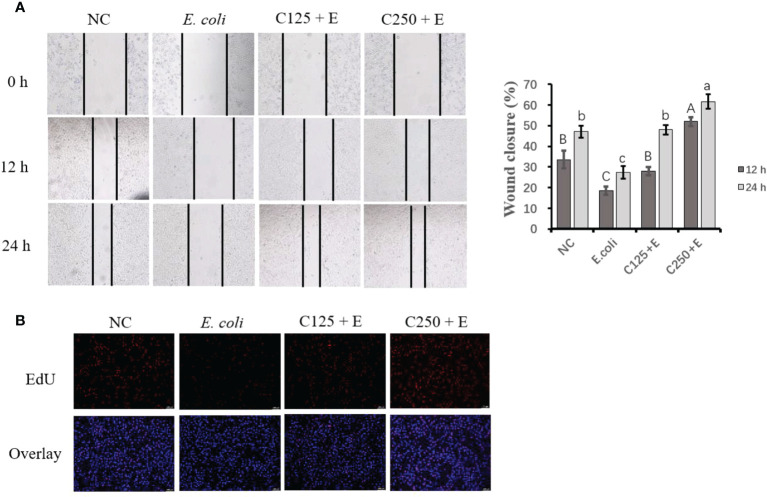
The effect of CA pretreatment on cell proliferation followed by *Escherichia coli* B1 stimulation. **(A)** Wound healing assay was performed for the migration ability of bMEC; the closure rate was imaged at 0, 12, and 24 h after scraping. **(B)** EdU determination for cell proliferation. Cells were observed in ×200 magnification (scale bar = 100 μm). Cells were pretreated with CA at doses of 125 or 250 μg/ml for 24 h, followed by the induction of *E. coli* B1 for 6h. Results are expressed as means ± SEM. Letters in lowercase indicate difference between groups at 12 h, while uppercase letters indicate difference between groups at 24 h (*p* < 0.05). CA, caffeic acid; bMEC, bovine mammary epithelial cell; EdU, 5-ethynyl-2′-deoxyuridine.

To determine the proliferation activity of bMECs exposed to different treatments in the present study, we used scratch wound assays and EdU staining (**Figure 3**). *E. coli* infection inhibited cell growth by reducing cell migration at both 12 and 24 h. Pretreatment with CA at 125 μg/ml restored less cell activity than at 250 μg/ml, which indicated that the effect of CA on bMEC proliferation is dose dependent. The results of the EdU assay consistently showed that pretreatment with CA at 125 or 250 μg/ml displayed strengthened EdU staining as compared to the *E. coli* group (*p* < 0.05), and the effect was dose-dependent.

### Pretreatment with caffeic acid inhibited proinflammatory responses in bovine mammary epithelial cells induced by *Escherichia coli*

**Figure 4:**
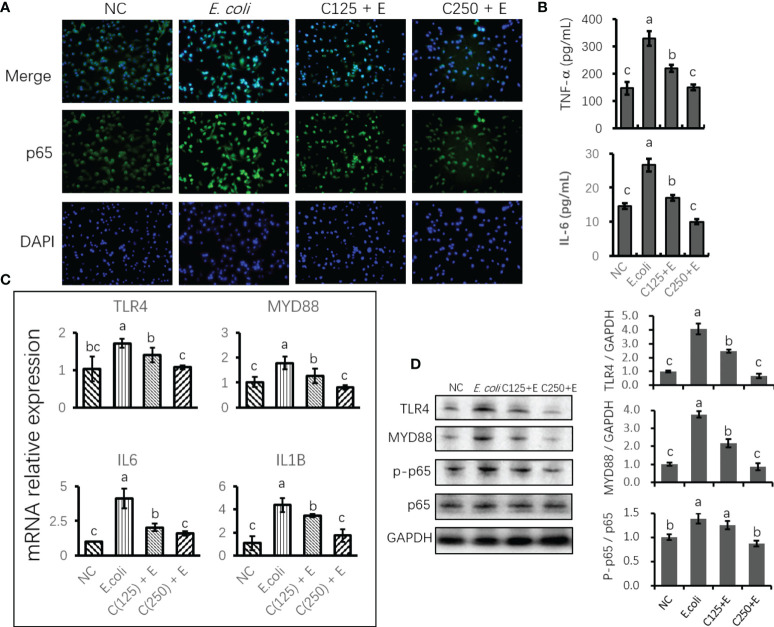
The effect of CA pretreatment on *Escherichia coli*-induced proinflammatory signaling components. **(A)** Immunofluorescence of p-p65 proteins expressed in bMECs and imaged using DMi8 Microsystems BmbH. Cells were observed at ×200 magnification. **(B)** Determination of the concentration of TNF-α and IL-6 in cultured medium using ELISA test. **(C)** The mRNA expression of TLR4 and NF-κB signaling genes. Abundance of each gene was normalized by the geometric mean of the internal control genes (GAPDH, UXT, and RPS9). Abundance of genes in the NC group was set as 1.0. **(D)** Immunoblots were determined for the expression of proteins related to TLR4 and NF-κB signaling. Experiments were performed as three independent replicates. Results are expressed as means ± SEM. Letters in superscript indicate that the difference between groups was significant (*p* < 0.05). CA, caffeic acid; bMECs, bovine mammary epithelial cells; NC, negative control.

To uncover the protective effect of CA on responses to *E. coli* stimulation in bMECs, we determined the expression of mRNA and proteins related to proinflammation. Cells infected with *E. coli* upregulated the mRNA expression of *TLR4*, *MYD88*, *IL6*, and *IL1B*. However, pretreatment with CA at 125 or 250 μg/ml significantly attenuated the expression of these genes as compared to those in the *E. coli* group (*p* < 0.05) (**Figure 4C**). Moreover, the expression of proteins related to TLR4 signaling was in agreement with the results of our gene expression determination. Meanwhile, an ELISA verified the downstream factors that are regulated by TLR4/NF-κB signaling, and the increased secretion of TNF-α and IL-6 in the cultured medium was inhibited by pretreatment with CA at 125 or 250 μg/ml (*p* < 0.05) (**Figure 4B**). Notably, the elevated ratio of phosphorylated p65 to total p65 in the *E. coli* group was lowered by the CA pretreatment at 125 or 250 μg/ml in a dose-dependent manner (**Figure 4D**). The results consistently showed that cells pretreated with CA at 125 or 250 μg/ml, followed by *E. coli* infection, weakened the staining of p65 as compared to that of the *E. coli* group (**Figure 4A**).

### *Escherichia coli*-induced oxidative stress in bovine mammary epithelial cells was attenuated by caffeic acid pretreatment

**Figure 5:**
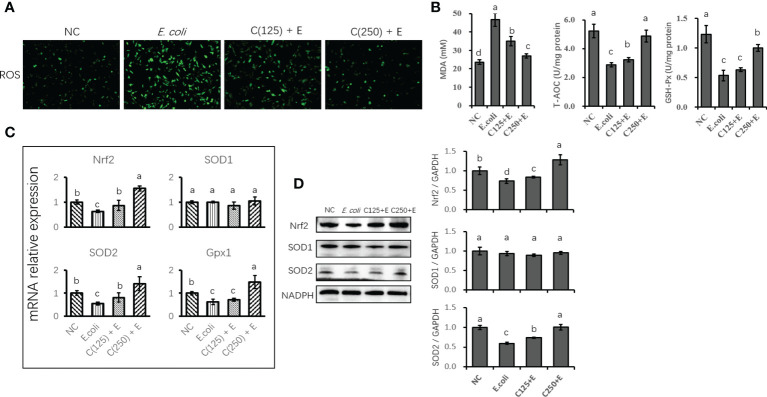
The antioxidative effect of CA pretreatment on cells stimulated with *Escherichia coli.* **(A)** DCFH-DA probe for intracellular ROS content was captured and determined. Cells were observed in a ×200 magnification. **(B)** The absorbance of intensity for MDA, GSH-Px, and T-AOC was obtained at 532, 420, and 520 nm, respectively. **(C)** The expression of genes related to antioxidants and Nrf2 signaling. Abundance of each gene was normalized by the geometric mean of the internal control genes (GAPDH, UXT, and RPS9). Abundance of genes in NC group was set as 1.0. **(D)** Immunoblots were determined for the expression of proteins related to antioxidants and Nrf2 signaling. Experiments were performed independently in triplicate. Results are expressed as means ± SEM. Letters in superscript indicate that the difference between groups was significant (*p* < 0.05). CA, caffeic acid; DCFH-DA, 2′,7′-dichlorofluorescein diacetate; ROS, reactive oxygen species; MDA, malondialdehyde; GSH-Px, glutathione peroxidase; T-AOC, total antioxidative capacity; NC, negative control.

The *E. coli*-induced production of ROS in bMECs was demonstrated by the increased staining of intracellular ROS in the *E. coli* group (**Figure 5A**). However, ROS production was inhibited by CA supplementation as a result of reduced staining in cells pretreated with CA at 125 or 250 μg/ml. Similarly, *E. coli* stimulation enhanced the concentration of MDA, and pretreatment with 125 and 250 μg/ml of CA significantly suppressed the accumulation of MDA as compared to the *E. coli* group. Regarding the antioxidative capacity (**Figure 5B**), GSH-Px and T-AOC activity were induced by *E. coli* infection, whereas CA pretreatment improved the antioxidant enzyme activity by increasing GSH-Px and T-AOC activity compared to the *E. coli* group.

The expression of genes related to antioxidants was determined using qPCR (**Figure 5C**). *E. coli* infection destroyed the antioxidant capacity of bMECs by reducing the expression of antioxidant enzyme-encoding genes (Nrf2 and SOD2). Pretreatment with CA at 125 or 250 μg/ml reversed the suppression of Nrf2 and SOD2 gene expression. However, SOD1 gene expression was not affected by either *E. coli* stimulation or CA supplementation as compared to the control group (*p* < 0.05). Additionally, the protein expression of Nrf2 and SOD2 was consistent with the gene expression compared to the results of the reversed downregulation of Nrf2 and SOD2 induced by *E. coli* infection (**Figure 5D**).

### Caffeic acid protected bovine mammary epithelial cells from the detrimental effects of *Escherichia coli* infection on lipid homeostasis

**Figure 6:**
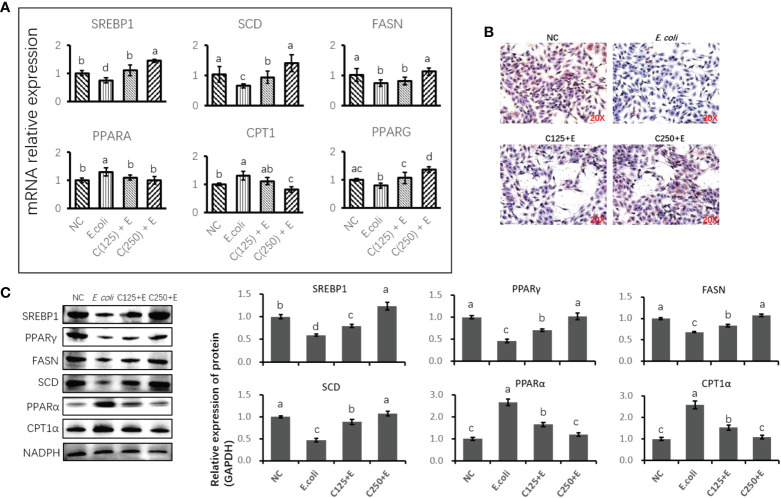
The effect of CA pretreatment on lipid metabolism in bMECs challenged by *Escherichia coli.* **(A)** The expression of genes related to lipid metabolism. Abundance of each gene was normalized by the geometric mean of the internal control genes (GAPDH, UXT, and RPS9). Abundance of genes in NC group was set as 1.0. **(B)** Lipid accumulation in bMECs by using oil red O staining assay. Cells were observed in ×200 magnification. **(C)** Immunoblots were determined for the expression of proteins related to lipid metabolism. Experiments were performed independently in triplicate. Results are expressed as means ± SEM. Letters in superscript indicate that the difference between groups was significant (*p* < 0.05). CA, caffeic acid; bMECs, bovine mammary epithelial cells; NC, negative control.

We further studied the effect of CA on the *E. coli*-induced alteration of lipid metabolism (**Figure 6**). The expression of genes and proteins related to fatty acid anabolism was downregulated by *E. coli* infection, including SREBP1, SCD, FAS, and PPARγ. However, pretreatment with CA at 125 or 250 μg/ml reversed the suppression of lipogenic genes and proteins induced by *E. coli* in a dose-dependent manner. In contrast, genes and proteins associated with lipid catabolism were upregulated by the *E. coli* infection, whereas pretreatment with CA inhibited the expression of PPARα and CPT1, as compared to the *E. coli* group in **Figures 6A, C** (*p* < 0.05). Regarding the secretion of cellular lipids in **Figure 6B**, *E. coli* infection in bMECs abolished lipid production in relation to oil red O staining, as compared to the control group (*p* < 0.05). In contrast, bMECs pretreated with CA at 125 and 250 μg/ml recovered their lipid synthesis following *E. coli* stimulation.

### Mouse lethality and histological alterations in mammary gland

**Figure 7:**
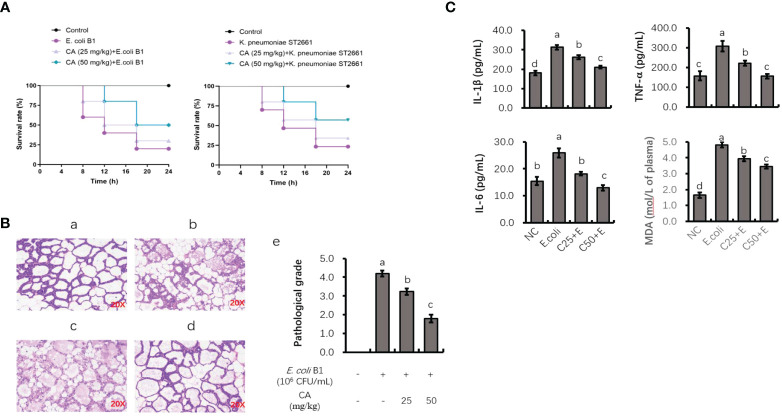
The protective role of CA on pathogenic bacteria-induced lethality and mammary tissue damage. **(A)** Mortality of mice administrated with CA followed by *Escherichia coli* or *Klebsiella pneumoniae* challenge. Mice were intraperitoneally injected with *E. coli* or *K. pneumoniae*, with oral administration of CA at 25 and 50 mg/kg for 10 days prior to *E. coli* or *K. pneumoniae* injection. **(B)** H&E staining of the mammary tissue after *E. coli* injection. Images from each experimental group were original magnification by ×200. (a) Control group; (b) mice treated with intramammary *E. coli*; (c, d) CA treated (25 and 50 m/kg) followed by *E. coli* injection; (e) evaluation of pathological grade. **(C)** Concentrations of IL-1β, TNF-α, and IL-6 in mice mammary tissue and peripheral content of MDA. Letters in superscript indicate that the difference between groups was significant (*p* < 0.05). CA, caffeic acid; MDA, malondialdehyde.

As shown in **Figure 7A**, the injection of *E. coli* or *K. pneumoniae* induced an 80% murine mortality rate within 18 h. However, pretreatment with CA at 50 mg/kg before an *E. coli* or *K. pneumoniae* injection significantly enhanced the viability of mice, with an increased survival rate of 30% or 40% as compared to the infected groups (**Figure 7A**). Regarding histological changes in mammary tissue (**Figure 7B**), the *E. coli*-infected group displayed evident histopathologic abnormalities as compared to the control group, including acinar congestion, inflammatory cell infiltration, and flocculus injury. In contrast, pretreatment with CA at 25 or 50 mg/kg significantly alleviated the histological changes induced by *E. coli*. Histopathological changes in the mammary glands were evaluated according to their injury degree score, including the integrity of mammary tissues and the infiltrated inflammatory cells. The pathological score was evaluated on a scale of 0–5, as described previously (40).

### Analysis of TNF-α, IL-1β, and IL-6 levels in mouse mammary tissue and malondialdehyde content in peripheral plasma

To investigate the anti-inflammatory role of CA *in vivo*, the effects of CA on the *E. coli* B1-induced production of proinflammatory cytokines (TNF-α, IL-1β, and IL-6) in mammary tissue were studied (**Figure 7C**). The production of TNF-α, IL-1β, and IL-6 was significantly elevated in the *E. coli*-infected group, whereas mice administrated with CA at both 25 and 50 mg/kg reversed the production of these cytokines in a dose-dependent manner (*p* < 0.05). Meanwhile, CA supplementation suppressed the content of MDA in peripheral plasma, which was increased in the *E. coli*-infected mice.

## Discussion

The sharp increase in bovine mastitis associated with MDR bacterial pathogens has become a serious issue for the dairy industry and a threat to public health, especially with respect to Gram-negative pathogens. Therefore, developing alternative strategies other than antibiotics to interfere with interactions between pathogenic bacteria and host-cell immune responses is a promising approach to circumvent this problem. The present study investigated the protective effects of CA in terms of antibacterial activities and immune-boosting bMECs. CA eradicates bacterial activity by disrupting both biofilm formation and the adhesion/internalization of the host’s epithelial cells. Meanwhile, CA supplementation enhances the host cell’s defenses by deactivating inflammatory and oxidative responses, as well as reprogramming lipid homeostasis in bMECs infected with *E. coli* B1. In addition, we demonstrated that the administration of CA in mice is effective in combating mammary tissue damage and systemic inflammation induced by clinically isolated Gram-negative bacterial infections.

Caffeic acid and its derivatives have been found to exhibit antimicrobial activity toward a diverse range of pathogenic microorganisms, including Gram-negative and Gram-positive bacteria. For instance, CA potentiates an antibacterial effect on *S. aureus* strains isolated from infected wounds alone and in combination with antibiotics–phytochemicals with a MIC range of 256–1,024 μg/ml (41). In addition, phenolic compounds, including CA, have been suggested to exert antimicrobial activity by inhibiting Enterobacteriaceae growth in the digestive tract (41). However, the preventive and therapeutic potential of CA in bacterial infections, such as bovine mastitis, is still elusive. In the present study, we selected prevalent strains isolated from milk produced by cows with clinical bovine mastitis to determine the MIC and MBC of CA. As stated in a previous study, *E. coli* and *K. pneumoniae* are the most isolated bacteria in Chinese farms (12), and the MIC and MBC values for MDR *E. coli* and *K. pneumoniae* in this study indicate that CA is effective in inhibiting Gram-negative MDR bacteria. Moreover, the growth curves validate the inhibitory effects of CA on the activities of *E. coli* and *K. pneumoniae*. However, a better understanding of the pharmacological mechanisms is necessary to determine which CA acts as an antibacterial agent.

It is notable that antimicrobial actions mainly occur when changing the permeability of cell membranes, enzyme activity, biofilm formation, or the structure of proteins and DNA, as well as blocking the adhesion or internalization of host cells. In the case of bovine mastitis infections caused by pathogenic bacteria, biofilm penetrating the teat and sticking to the internal surface of mammary tissue is suggested to help develop MDR in *E. coli* strains (42, 43). Therefore, taking into consideration the blocking of biofilm formation to potentiate the elimination of troublesome pathogens is one way of finding alternatives to antibacterial agents. In this study, a 2× MIC of CA was found to curb the formation of biofilm and eradicate already formed, mature biofilm by killing bacteria. Therefore, when considering the increasing emergence of MDR bacteria, CA is an agent that can reduce the difficulty of using antibiotic treatments. However, unlike other studies, we found that combination therapies using CA and antimicrobial agents such as cephalothin, imipenem, lincomycin, and penicillin showed no synergetic effect on *E. coli* inhibition (data not shown).

Except for biofilm protection, the adhesion/internalization of extracellular bacteria such as *E. coli* is believed to enhance bacterial survival by increasing antibiotic tolerance in the host cell’s cytosol (26, 44). As such, the inhibited adhesion or internalization of *E. coli* caused by increasing the amount of CA indicates that CA is capable of reducing interactions between *E. coli* and bMECs and, therefore, the invasion of cells. However, the mechanism by which CA blocks the adherence properties of pathogenic bacteria in epithelial cells needs to be further investigated.

Generally, the host triggers inflammatory responses to reduce the detrimental effects induced by pathogen-associated molecular patterns (PAMPs) such as LPS, the major constituent of Gram-negative bacteria walls. *E. coli* strains usually cause acute coliform mastitis, and they play an important role in mammary epithelial cell impairment when the bacteria are lysed by immune cells such as phagocytes. To detect signs of proinflammatory molecule changes, the components of the TLR4/NF-κB signaling pathway can be monitored by using an *E. coli* B1-infected cell model. In the present study, the supplementation of CA downregulated TLR4/NF-κB signaling components induced by *E. coli* B1 infection, which is consistent with downstream-regulated cytokine cascades such as IL-6 and TNF-α. Moreover, it has been reported that CA can prevent LPS-induced bMEC inflammatory injuries *via* NF-κB and MAPK activation (30). Along with its role in prohibiting the translocation of p65 into the nucleus, the current study additionally validated the role of CA in protecting bMECs from pathogen-directed infections by inhibiting TLR4/NF-κB signaling.

Oxidative stress-induced tissue injuries manifest as Nrf2 signaling activation due to interactions with NF-κB-mediated inflammation. Accumulated levels of oxygen-free radicals, such as ROS, and assessments of the antioxidant capacity of cells are used as indicators of oxidative damage. During LPS-induced acute phase responses, macrophages sensitive to the LPS-induced generation of ROS seem to be regulated by the MAPK and NF-κB transduction pathways. Similarly, bMECs and macrophages infected with extended-spectrum beta-lactamase (ESBL) *E. coli* have been reported to show signs of oxidative stress *via* their interactions with TLR4/NF-κB pathway (45). In the present study, the decreased capacity of antioxidants caused by stimulating bMECs with *E. coli* B1 was reversed using a CA pretreatment, as indicated by changes in antioxidative enzyme activities. Hence, the result is in agreement with studies finding that CA is a viable alternative for ameliorating a wide spectrum of disorders with its anti-inflammatory and antioxidation properties (46).

As one of the main functions of bMECs is the production of milk fat, we evaluated the regulatory effect of CA on the *E. coli* B1-induced imbalance of fatty acid metabolism. The intracellular content of lipids refers to the accumulation of packaged lipid droplets, which are the precursors of the fat that is secreted into milk. In the present study, expressions of genes and proteins related to fatty acid synthesis were downregulated, whereas the expression of fatty acid oxidation-associated genes and proteins was upregulated by *E. coli* infection. Lipid metabolism in bMECs infected with *E. coli* appears to switch from fatty acid anabolism to catabolism since activating the inflammatory and acute phase responses requires significant amounts of energy. Moreover, in conditions of pathogenic bacteria infection, lipogenesis and lipolysis indicate an inverse relationship in bMECs between the immune response and milk fat synthesis (47). The present study reveals that CA pretreatment is capable of controlling the lipid homeostasis of bMECs, as well as restoring lipogenesis, which is impeded by the stimulation of pathogens. Collectively, this provides evidence that CA promotes lipid synthesis along with a decrease in lipid oxidation, which may contribute to the detoxification of oxidative stress and a reduction in the inflammatory responses induced by *E. coli* B1 infection.

Since the efficacy of CA’s antimicrobial and protective effects in host bMECs was validated *in vitro*, we conducted an *in vivo* experiment by administrating CA and *E. coli* or *K. pneumoniae* to mice in order to determine survival rates. Infecting mice with *E. coli* or *K. pneumoniae* strains led to a 20% mortality rate; however, the survival rate was elevated by the administration of CA. Clinically isolated *E. coli* strains induce murine mastitis, which is believed to elicit histological changes along with the elevated expression of proinflammatory genes (48). After injecting *E. coli* or *K. pneumoniae* strains into the lactating mammary tissue of mice, serious damage to histological integrity induced by this pathogenic infection was consistently treated by CA administration. Moreover, the acute phase responses concurred with the elevated cytokines in the mammary tissue, and MDA content in peripheral plasma was attenuated by administrating CA. These results indicate that CA can effectively reduce inflammation in mouse mammary glands induced by Gram-negative bacteria.

In summary, versatile CA possesses antimicrobial and immune-boosting properties, coupled with an ability to interfere with interactions between pathogenesis and host-cell responses, suggesting that it represents a promising alternative to tackle pathogenic bacteria-infected bovine mastitis. Moreover, the verification of CA in the development of potential therapeutic agents for bovine mastitis will encourage work into uncovering more candidates for non-antibiotic treatment. Nevertheless, more clinical trials are necessary to verify the potential activity of CA in the treatment of bovine mastitis.

## Data availability statement

The datasets presented in this study can be found in online repositories. The names of the repository/repositories and accession number(s) can be found in the article/**Supplementary Material**.

## Ethics statement

This study was reviewed and approved by the Animal Experiment Committee of Yangzhou University.

## Author contributions

TX and ZY provided the study concept and design. ZY and TX developed the methodology and wrote, reviewed, and revised the paper. TX, GC and RL acquired, analyzed, and interpreted the data and performed statistical analysis. HZ and YY provided technical and material support. All authors contributed to the article and approved the submitted version.

## Funding

This study was supported by the National Natural Science Foundation of China (32102731, 31872324) and the Open Project Program of Joint International Research Laboratory of Agriculture and Agri-Product Safety, the Ministry of Education of China, Yangzhou University (JILAR-KF202204).

## Conflict of interest

The authors declare that the research was conducted in the absence of any commercial or financial relationships that could be construed as a potential conflict of interest.

## Publisher’s note

All claims expressed in this article are solely those of the authors and do not necessarily represent those of their affiliated organizations, or those of the publisher, the editors and the reviewers. Any product that may be evaluated in this article, or claim that may be made by its manufacturer, is not guaranteed or endorsed by the publisher.
