# Supplementary material for: The protective role of caffeic acid on bovine mammary epithelial cells and the inhibition of growth and biofilm formation of Gram-negative bacteria isolated from clinical mastitis milk
Source: Front Immunol. 2022 Oct 20;13:1005430. doi: 10.3389/fimmu.2022.1005430 (PMC9632277; doi:10.3389/fimmu.2022.1005430)
Supplement: Supplementary file 1 [file DataSheet_1.pdf]

The protective role of caffeic acid on bovine mammary epithelial cells and inhibition of gram-negative bacteria isolated from clinical mastitis milk

Tianle Xu[1,2], Hao Zhu[2], Run Liu[2], Xinyue Wu[2], Guangjun Chang[4], Yi Yang[3], Zhangping Yang[1,2*]

[1] Joint International Research Laboratory of Agriculture and Agri-Product Safety, Ministry of Education of China, Yangzhou University, Yangzhou 225009, China;

[2] College of Animal Science and Technology, Yangzhou University, Yangzhou 225009, China;

[3] College of Veterinary Medicine, Yangzhou University, Yangzhou 225009, China;

[4] College of Veterinary Medicine, Nanjing Agricultural University, Nanjing 210095, China.

* Correspondence: yzp@yzu.edu.cn; Tel.: +86-(51)-487977307

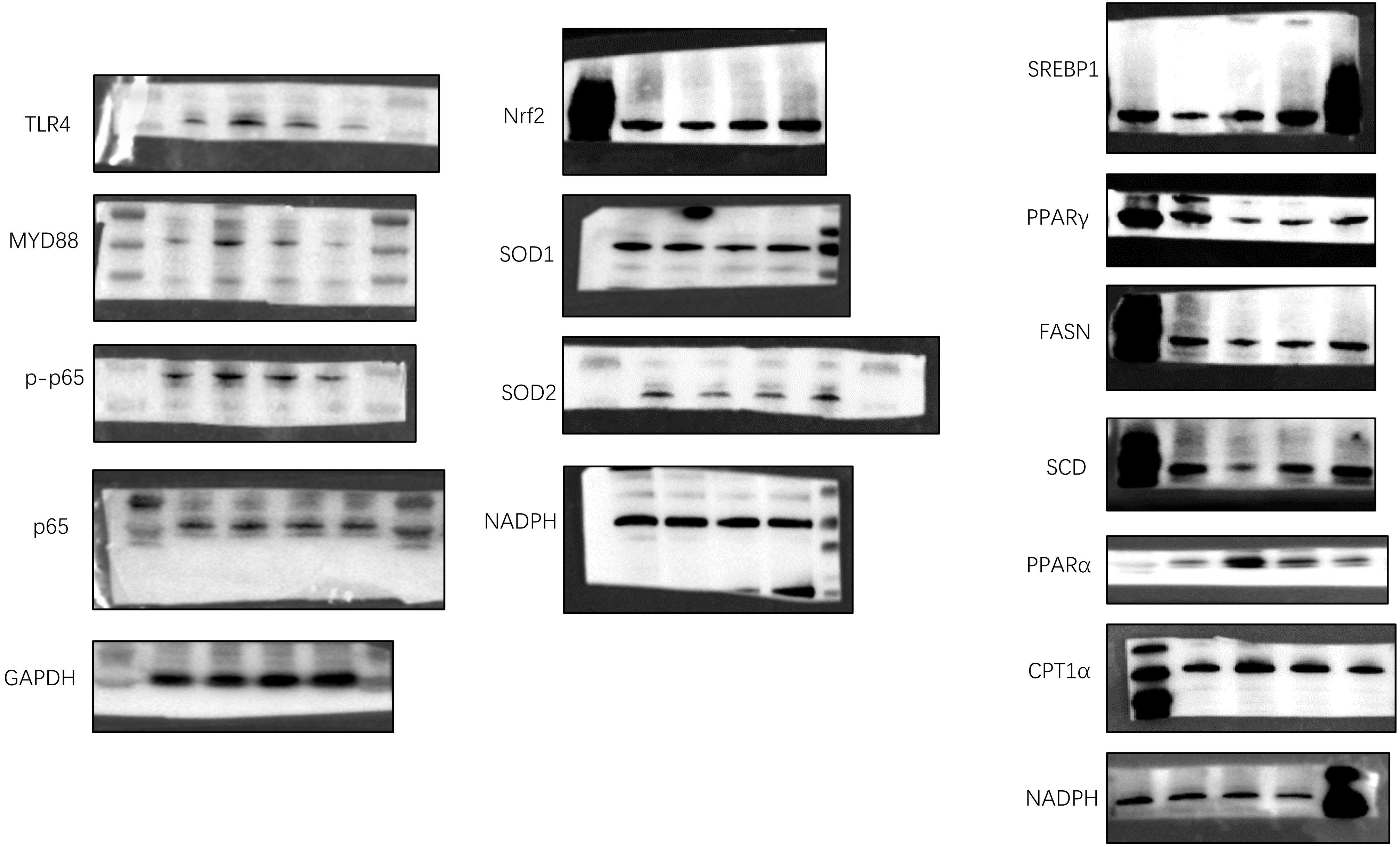

TLR4
MYD88
p-p65
p65
GAPDH
Nrf2
SOD1
SOD2
NADPH
SREBP1
PPARγ
FASN
SCD
PPARα
CPT1α
NADPH
