# Supplementary material for: The protective role of caffeic acid on bovine mammary epithelial cells and the inhibition of growth and biofilm formation of Gram-negative bacteria isolated from clinical mastitis milk
Source: Front Immunol. 2022 Oct 20;13:1005430. doi: 10.3389/fimmu.2022.1005430 (PMC9632277; doi:10.3389/fimmu.2022.1005430)
Supplement: Supplementary file 2 [file DataSheet_2.docx]

**Supporting information**

**The protective role of caffeic acid on bovine mammary epithelial cells and inhibition of gram-negative bacteria isolated from clinical mastitis milk**

Tianle Xu^1,2^, Hao Zhu^2^, Run Liu^2^, Xinyue Wu^2^, Guangjun Chang^4^, Yi Yang^3^, Zhangping Yang^1,2*^

^1^ Joint International Research Laboratory of Agriculture and Agri-Product Safety,

Ministry of Education of China, Yangzhou University, Yangzhou 225009, China;

^2^ College of Animal Science and Technology, Yangzhou University, Yangzhou 225009, China;

^3^ College of Veterinary Medicine, Yangzhou University, Yangzhou 225009, China;

^4^ College of Veterinary Medicine, Nanjing Agricultural University, Nanjing 210095, China.

* Correspondence: yzp@yzu.edu.cn; Tel.: +86-(51)-487977307

**Milk samples**

A total of 289 quarter-milk samples from mastitis-infected cows (with positive result using California Mastitis Test and a somatic cell count > 400,000 cells/mL) were randomly collected from four major dairy farms in Jiangsu province of China, including Northern Jiangsu (n=186, Sihong and Xuyi), Mid Jiangsu (n=48, Gaoyou), Southern Jiangsu (n=55, Zhangjiagang). The samples were continuously collected from Spring (March) to Autumn (September) in 2020 over two seasons. All milk samples (50-mL for each quarter) were proceeded within 24 h for experiment. The experimental procedures in the current study used for cows and mice were approved by the Animal Experiment Committee of Yangzhou University (YZU202002-153). All experimental protocols were performed in accordance with approved guidelines and regulations.

**Isolation and identification of bacterial strains**

The isolation and identification of bacterial were conducted as recommended by the U.S. National Mastitis Council[1; 2]. In brief, the milk samples were plated onto blood agar supplemented with 5% fresh sheep whole blood and incubated at 37°C aerobically for 24 h. Based on the morphology of colonies, single identical colony of each sample was then sub-cultured by streaking on Luria Broth (LB) agar. Plate-cultured bacterial were expanded in nutrient broth at 37°C aerobically for 24 h. All the suspected isolates were further confirmed via 16S rDNA sequencing[3]. The confirmed isolates were kept in 15% glycerol at -80 °C as frozen stock. A PCR method were performed to rapidly determine the phylogenetic groups of *E. coli* strains into four main groups (A, B1, B2 and D) according to the positive expression of three reference genes (chuA, yjaA, TspE4.C2) as described[4]. For multilocus sequence typing (MLST) analysis, strains including E. coli, K. pneumoniae, S. aureus, P. aeruginosa were determined by the corresponding housekeeping genes and subsequently blast on the PubMLST (<https://pubmlst.org/>). The most isolated subs trains were taken into the determination of MIC or MBC for CA efficacy.

[1] J.S. Hogan, R.N. Gonzalez, R.J. Harmon, S.C. Nickerson, and K.L. Smith, Laboratory handbook on bovine mastitis. Madison, WI: National Mastitis Council. (1999).

[2] S. Blum, E.D. Heller, O. Krifucks, S. Sela, O. Hammer-Muntz, and G. Leitner, Identification of a bovine mastitis Escherichia coli subset. Veterinary Microbiology 132 (2008) 135-148.

[3] J.A. Frank, C.I. Reich, S. Sharma, J.S. Weisbaum, B.A. Wilson, and G.J. Olsen, Critical evaluation of two primers commonly used for amplification of bacterial 16S rRNA genes. Appl Environ Microbiol 74 (2008) 2461-70.

[4] O. Clermont, S. Bonacorsi, and E. Bingen, Rapid and simple determination of the Escherichia coli phylogenetic group. Appl Environ Microbiol 66 (2000) 4555-8.
